# Placement Stability, Cumulative Time in Care, and Permanency: Using Administrative Data from CPS to Track Placement Trajectories

**DOI:** 10.3390/ijerph14111405

**Published:** 2017-11-17

**Authors:** Sonia Hélie, Marie-Andrée Poirier, Tonino Esposito, Daniel Turcotte

**Affiliations:** 1 Research Institute for Youth in Difficulty, University Health and Social Services Center-South Central Montreal, 1001, boulevard de Maisonneuve Est, Montreal, QC H2L 4R5, Canada; 2 School of Social Work, University of Montreal, 3150, Jean-Brillant, Montreal, QC H3C 3J7, Canada; marie-andree.poirier@umontreal.ca; 3 Canada Research Chair in Social Services for Vulnerable Children, School of Social Work, University of Montreal, 3150, Jean-Brillant, Montreal, QC H3C 3J7, Canada; tonino.esposito@umontreal.ca; 4 School of Social Work, Laval University, 1030, avenue des Sciences Humaines, Quebec, QC G1V 0A6, Canada; daniel.turcotte@svs.ulaval.ca

**Keywords:** foster care, placement, out-of-home care, stability, permanency, child welfare, protection services, administrative data

## Abstract

*Objectives*: The Quebec Youth Protection Act was amended in 2007. The main goal of this reform was to improve placement stability for children who are removed from their home for their protection. Among several legal provisions introduced was the establishment of maximum age-specific durations of out-of-home care, after which a plan must be established to provide stability for children placed in substitute care by finding permanent homes for them. The purpose of this study is (1) to examine trends in placement use and placement stability since the reform and (2) to document the current frequency of each type of placement setting, the cumulative time in care before the exit to permanency, and the sustainability of the permanency outcome. *Methods:* The study relies on 3 entry cohorts of all children investigated who received protection measures in the province of Quebec during 3 specific time frames before and after the reform (*n* = 9620, 8676, 8425). Cohorts were observed for a period varying from 3 to 4 years. Administrative data from all 16 child protection agencies were used to track placement trajectory indicators and to compare cohorts. *Results*: There has been a decrease in the proportion of children receiving protection measures who were placed in care since the reform, and placement in kinship care has become more frequent among children placed. Placement stability improved slightly after the reform. Overall, for infants, the most frequent type of permanency attained is adoption, while reunification is the option most often indicated for older children. Some children are at a greater risk of experiencing unstable placement trajectories: young children have a high rate of reunification breakdown, some wait a long time to be adopted, and adolescents are frequently removed from the substitute care setting where they were supposed to stay until the age of 18. *Conclusions*: The results suggest interesting avenues for policy makers and service providers to improve the stability of placement trajectories. Advantages and disadvantages of administrative data are discussed.

## 1. Introduction

Major amendments were made to the Quebec Youth Protection Act (YPA) in 2007. The main goal of the reform was to improve the stability of children in care by reducing the number of times they were moved from one substitute home to another. While the stability of placements and permanency planning have always been part of the YPA, a systematic and reliable examination of these dimensions and their evolution has never been done for the whole province. Obviously, in some cases, a move from one home to another may improve a child’s welfare [1], especially if it results in a setting that is a better fit. Yet, attachment theory—which suggests that it is essential for children to foster the development of stable emotional bonds—has had an unequivocal influence on the introduction of maximum stays in care in various CPS systems. For a long time, authors pointed out that major, as major psychological damage can be caused by repeated relational disruptions, particularly at an early age [2,3]. The principles set out in the new provisions of the YPA reaffirmed that every decision made by child protective services (CPS) must seek to keep children in the family environment and return them to their homes as soon as possible if they have to be removed. The Act also states that “(i) f, in the interest of the child, returning the child to the family is impossible, the decision must aim at ensuring continuity of care, stable relationships and stable living conditions corresponding to the child’s needs and age on a permanent basis” (s. 4). A number of provisions were introduced to promote permanency, including a requirement for systematic consideration of kinship care and maximum lengths of stays in care by age, after which the court must make a ruling to provide permanent living conditions. The maximum stays are set at 12 months if the child is under 2 years of age, 18 months if the child is from 2 to 5 years of age, and 24 months if the child is 6 years of age or over. In addition, under the new provisions in section 156.2 of the YPA, the Minister of Health and Social Services must periodically table a report in the National Assembly measuring the impact of the Act on various aspects of the placement trajectory. Pursuant to this section, two evaluative studies have been conducted since the reform. The first one looked at the changes that took place in the first few years following the reform [4,5,6]. Those findings were consistent with the goal of the new YPA. They revealed that out-of-home care is less frequent, the instability of placements observed over a two-year period declined slightly, and that kinship care is increasingly common. The present study is the second assessment done since the reform. It has two aims. The first is to determine whether the changes observed in the first study have been maintained over a longer observation period of three to four years. The second, and more important, is to paint a preliminary picture of the types of permanency attained by children in care and the cumulative length of stay in substitute care before permanency is attained. This study’s novel contribution is an overview of the placement trajectory, from the first spell in out-of-home care until the latest exit to permanency, covering all the successive service spells occurring within the observation period. It proposes a broad definition of placement and a number of stability indicators based on CPS administrative data. In the context of this study, we consider that instability occurs as soon as a child is moved from one substitute care setting to another. Although permanency usually refers to a living arrangement that is legally permanent for the child, such as reunification, adoption or guardianship, additional options are examined in the current study and described below. Also, many dimensions have been included in the concept of placement trajectory in order to encompass the main events that punctuate the child’s comings and goings through different living environments: placement, type of substitute care setting, stability of placement, cumulative length of stay in care, type of permanency achieved, and it’s sustainability.

### 1.1. Placement, Kinship Care and Stability

The study of a Quebec clinical population by Esposito and colleagues found that 23% of children investigated by CPS were placed in out-of-home care [7,8]. When only the children who received protection measures are considered, the percentage of placements is necessarily higher. Indeed, official CPS statistics report that 52% of children under CPS supervision were placed in care as of 31 March 2015 [9]. Not only is this figure is a cross-sectional measurement, it also excludes emergency and temporary placements. The definition of placement used in the evaluation of impact of the YPA is broader and counts any removal of a child from the home, for any length of time, regardless of whether the removal is planned or done on an emergency basis, and regardless of whether it is temporary or permanent [4,5]. Hélie et al. (2011) reported that before the reform, 63% of children receiving protective services were placed in out-of-home care within two years of intake into the CPS system. The proportion went down to 59% for children taken into the system right after the reform [5]. Few countries with comparable child protection system publish official and reliable statistics on placement use, however American and Australian reports provide points of comparison. In 2015, 23% of American children investigated by child protective services were taken into care after the investigation [10]. In Australia, 89% of all children under CPS supervision as of 30 June 2016, were placed in substitute care [11]. While official statistics from other countries may be helpful to contextualize placement use in the province of Quebec, the observed differences between countries are difficult to interpret. Legislation, definitions, clinical practices, resources allowed to child welfare and characteristics of the population are all factors that may explain, at least partly, these discrepancies.

Kinship care is currently, in Quebec and elsewhere, a trend partly attributable to the greater stability such placements represent [12,13,14,15,16,17,18,19,20]. Aside from stability, social, economic and political factors have stimulated the growing interest in kinship care: policies that emphasize keeping families together and approaches that focus on their strengths; overloaded official foster care systems; the need to preserve the cultural heritage, identity, and sense of belonging of children in care and; political philosophies that aim to lower the cost of public services [21]. In 2015, 30% of children in care in the U.S. were placed with kin [22]. In Australia, that same year, 49% of children in care were placed with kin [11]. The first evaluative study of the YPA found that not only is kinship care frequently used, but that its use is increasing. Using a comparable point in time measure, official statistics indicate that as of 31 March 2015, 20% of out-of-home children were placed in kinship care [9]. With a longitudinal measure, Hélie et al. (2011) found that before the reform, 25% of children entering care were placed with kin during the two following years, and afterwards, 33% [4,5].

Out-of-home care, although sometimes necessary, affects children and their families in many ways. Being taken into care changes children’s daily lives and transforms their relationships with friends and family. The transition may be stressful because it requires adjustments in a number of areas: changing schools [23], losing friends, fitting into a new household, getting used to a new neighborhood [24,25,26], and every subsequent move exacerbates this impact because it means another disruption. It is therefore not surprising that substitute care’s usefulness as a protective measure is sometimes called into question because it exposes children removed from their homes to instability, which may make them more vulnerable. Although not new, the concern for the stability of children in care has grown with the knowledge of the adverse effects that moves from one placement to another has on placed children [20]. Research indicates that children who have been moved around have more behavioral and emotional problems [27,28,29], academic problems [30], and have more trouble forging emotional bonds with parental figures [20,31,32]. It has also been found that being moved increases the risk of subsequent moves and reunification breakdown [32,33].

Despite this recognition, various studies have found far from negligible placement-change rates among children in care. Esposito et al. (2014) have reported on moves over an observation period of up to 9 years, for a clinical population of 29,040 Quebec children in care for the first time. They found that 31% of children were not moved at all during the observation period, 25% were moved just once, 16% were moved twice, and 28% were moved three or more times [8]. In England, one author found slightly higher rates, despite her shorter observation period of 3.5 years: 19% of children in care stayed in the same place, 22% were moved once, and 59% were moved two or more times [34]. Authors who have studied placement stability generally just count the number of moves within the same placement spell. But a child may be moved to a known substitute home or to new surroundings. For instance, a child may be moved back and forth several times between two different substitute homes. Furthermore, a child under CPS supervision may experience a number of placement spells within one or more service spells. Our study uses more exhaustive stability indicators. All the moves in all the placement spells within the observation window were included in a first stability indicator. Then a second indicator describing the number of different substitute homes the child was placed in during the observation period was calculated. Our first study, with a two year observation period, found that before the reform, 40% of children stayed in the same care setting and that after the reform, the proportion went up to 44% [5]. That study also revealed that in both study cohorts, the number of different substitute homes the child stayed in (an average of 2.06 in the Post cohort) was lower than the number of moves (an average of 2.23 in the Post cohort), which means that each new move was not necessarily to a substitute home unknown to the child; hence the importance of considering a number of stability indicators. In comparison, with an 18-month follow-up, one study found an average number of placements of 4.4 [35].

Generally speaking, whether in terms of placement, kinship care or stability, the results of Hélie et al.’s first evaluative study seem encouraging, even with the use of exhaustive indicators [4]. They suggest an improvement in the situation since the reform, at least in the first 2 years that children are under the supervision of child protective services. Now it remains to be determined whether the trend continues beyond the 26 months of that study’s observation period and for cohorts of children who entered the CPS system longer after the reform.

### 1.2. Cumulative Length of Stay, Type of Permanency, and Sustainability

In the literature, the duration of foster care is generally considered separately, depending on the type of exit to permanency. Most often, it is studied in relation to three types of permanency: family reunification, adoption and guardianship. It is hard to find clear trends in the literature with respect to the cumulative length of stay for each type of permanency, partly due to the considerably varying observation periods, which range from 12 months [36] to more than 9 years [37,38]. Concretely, the method used in most studies consists of identifying a cohort of children who begin a placement during a given time frame, often one or two fiscal years. The use of such entry cohorts, as opposed to exit cohorts and cross-sectional designs, is strongly recommended in child welfare outcomes research [39,40], especially when the objective is to track changes over time [41,42]. As stated by D’Andrade and colleagues (2008), “Exit cohorts are likely to be biased in important ways, since they exclude all youth who do not leave care. As a result, indicators derived from exit cohorts will tend to misrepresent the proportion of cases achieving permanency outcomes within the time frames” [39] (p. 146). The children’s placement trajectory is then observed to determine which of them are reunited with their families, adopted, or placed in guardianship, and to record how many days they were in care before their first exit to permanency. Of course, the longer a cohort’s observation period, the longer the median length of stay in substitute care are likely to be, because the calculation will include long-term placements. So with respect to reunification, studies that have observation periods shorter than two years report median stays of 30 to 366 days [39,40,41,42]. In those with longer observation periods (5 to 10 years), median stays in foster care prior to reunification varied from 175 to 415 days [37,38,43]. Stays in care before adoption were much longer, ranging from 737 days for a 30-month observation to 1678 days for a 10-year observation [36,38,43,44]. The cumulative length of stay in care is rarely documented in studies on guardianship. One study with a 30 to 42-month follow-up found that half of children who exited to guardianship were in out-of-home care for a total of more than 474 days [43] while in another study with a 5-year follow-up the median duration of stay was 704 days [45]. In the case of adoption and guardianship, the eligibility criteria and legal proceedings involved mean that there is a long preparatory period, which takes place in parallel with the protection process. It is largely for this reason that the cumulative length of stay in care before exiting to adoption is generally longer than the cumulative stay prior to reunification.

The concept of permanency described in these studies is nonetheless relative, because only the first exit to permanency is considered. It is quite possible that the first permanency goal attained may not be sustained over time and that the child must be placed once more. Take the case of a child returned home, but who must be removed again, only to have another reunification attempted. In this regard, re-entry studies demonstrated that these back and forth between family and foster home are not rare. Re-entry in care within 12 months following reunification varies between 8% and 16% [44,46,47]. With longer follow-ups of 7 to 10 years, the probability of re-entry varies between 20% and 37% [38,48,49]. In addition, all these events may either take place in the same service spell or be spread out over several. To get an accurate long-term idea of how much time is spent in care, the full history of services received by the child must be considered. It should also be kept in mind that permanency is relative, hence the need to count the number of unsuccessful attempts to attain permanency. A simple count of the total number of days spent in out-of-home care cannot reflect this complexity.

### 1.3. Objective

The overall objective of this study is to examine the changes in a number of aspects of children’s placement trajectories since the overhaul of the Quebec legislative framework, considering all the service spells children experienced during the observation period and using a variety of exhaustive indicators. More specifically, the primary aim of this study is to confirm whether the changes observed in the two years following the reform, in terms of placements, kinship care, and stability, are maintained over a longer observation period and in a cohort of children who have entered the CPS system more recently. Second, this study paints a preliminary picture of the types of permanency attained, cumulative length of stay in care before they are attained, and their sustainability.

## 2. Methods

This study received ethical clearance from all participating CPS agencies, through the research-ethics committee of the Centre intégré universitaire de santé et services sociaux de la Capitale Nationale.

### 2.1. Cohorts Studied

In this study, the placement trajectories of all children entering the Quebec CPS system at three different times were compared (Children living in two northern regions of Quebec are not included in the population under study, because at the time of this study, they were not served by a regular CPS agency, but rather through a specific community service center. Children in those regions constitute 0.7% of all the children in Quebec [9], many of which are Indigenous): four years before the coming into force of the amendments to the Youth Protection Act (Pre cohort), the year following its coming into effect (Post1 cohort), and the 18 months afterwards (Post2 cohort). Table 1 illustrates the eligibility period and observation period for each cohort. To establish each cohort, the following eligibility criteria were used (with different dates): the child must have been referred to and investigated by Quebec child protective services during the eligibility period and the child must have received protection measures. The children in the three cohorts had comparable observation periods, which were between three and four years, depending on when they entered the eligibility time frame. Children eligible for more than one cohort were included only in the first.

### 2.2. Data Source

The data come from the clinical/administrative databases of all the 16 Quebec CPS agencies. These agencies use the Projet Intégration Jeunesse (PIJ) client information system. CPS caseworkers add data to the system daily. Sociodemographic information on children and their parents are stored, along with the characteristics of situations reported and the services delivered under the YPA. Major efforts have been made to standardize both processes and content, so that the information held is standardized and validated [50].

### 2.3. Study Variables

The concept of placement (out-of-home or substitute care) refers to any removal of children from their homes, for any length of time, regardless of whether the removal is done on an emergency basis or is planned, nor whether it is temporary or permanent, or the type of care chosen. Kinship care is a dichotomous variable that indicates at least one placement with a member of the child’s extended family or friend during the observation period (the other possible types of substitute care are foster family, group home and rehabilitation center). In order to get a good idea of the stability experienced by children, two indicators have been used: (1) number of different substitute homes the child has stayed in and (2) number of moves directly from one substitute home to another. As presented above, prior work with these two indicators suggest that they are measuring different aspects of placement stability. Given that the information concerning kinship placements are not reliably documented in the client information system, these placements could not be included in the stability variables. As a result, only children with at least one non-kin placement (foster family, group home or rehabilitation center) are considered for the calculation of stability indicators and only the part of their trajectory that takes place in those settings is considered.

The cumulative length of stay in care before the latest exit to permanency was calculated by adding up the number of days between the starting date of the first placement noted in the client information system and the date of the latest permanency attained before the end of the observation period during which the child lived with non-kin. All types of placement were counted, for any length of time, regardless of whether the removal was done on an emergency basis or was planned, nor whether it was temporary or permanent, or whether it was voluntary or court ordered. The reason for the end of the last placement entered in the system determined the latest type of permanency attained. The types of permanency considered in the study were reunification, adoption, and placement in a given setting until the age of 18. Placement until the age of majority is not generally considered in the literature to be a permanent option, but as this choice is among the possibilities offered to Quebec CPS caseworkers to ensure the stability of children removed from their homes, we have included it here. Also, in Quebec, some adolescents may exit care for independent living and having been prepared for this before leaving care. This type of exit is documented in the current study as non permanent because the youth is leaving care without being legally connected, or reconnected to a family. An indicator of lack of sustainability of the latest permanency attained was established for the children whose latest life plan goal was attained at least six months before the end of the observation period. Lack of sustainability is defined as the start of a new placement after the latest exit to permanency. For children whose latest exit was reunification, lack of sustainability refers to a re-entry in care post-reunification. For children whose latest exit was to placement until 18, lack of sustainability means that a new placement took effect in a new setting more than one week after the court order. For children whose latest exit was to independent living, lack of sustainability refers to any placement occurring after the last exit. Finally, the number of exits to permanency during the observation period was also calculated, regardless of whether they were sustained.

Cumulative length of stay, the latest type of permanency attained, the lack of sustainability and the number of exits to permanency were calculated for an individual subset of the cohorts studied. First, the client information system data used to calculate the total length of stay and measure the types of permanency that were not available before the reform. As the Post2 cohort is more recent and the time elapsed between the reform and the cohort’s eligibility window is longer, that allowed more time for the new provisions of the YPA to be implemented and therefore the cumulative length of stay and type of permanency could be examined beginning with that cohort. Second, the maximum placement stays prescribed in the reform apply only to children whose cases go before the courts. As a result, only children in the Post2 cohort whose cases went before the courts and who were placed with non-kin before the end of the observation period, 31 December 2012, are included in these findings (*n* = 2280). Of this number, 174 children had invalid data on the placement dates used to calculate duration. The study group for the indicators of cumulative length of stay and type of permanency was therefore 2106 children.

Additional variables were used to describe the sociodemographic characteristics of the children in each cohort and some information on the investigated cases. Age of the child at entry in the cohort is computed as a continuous and a categorical variable. In both cases, it corresponds to the age of the child at the beginning of the investigation targeted in the study. Gender of the child is coded as boy or girl. Indigenous status is a dichotomic variable indicating if the child is from aboriginal descent. There may be several grounds for CPS intervention. The presence of each of the following ground for intervention was coded as a dichotomy: neglect, serious risk of neglect, physical abuse, serious risk of physical abuse, sexual abuse, serious risk of sexual abuse, serious behavioral disturbance, abandonment, and emotional maltreatment. The conclusion of the investigation targeted in the study indicate if the allegations are founded, and if the safety or development of the child is endangered by the reported situation. It can take one of the following values: founded allegations with safety or development endangered; founded allegations but safety or development not endangered; and unfounded allegations. Court use is a dichotomic variable that refers to the presence of at least one protection measure ordered by the court (as opposed to measures that are applied by voluntary agreement between the child/family and CPS). The length of services is the number of months between the entry and the termination of the last CPS spell, during which the CPS case of the child is active. The number of CPS episodes refer to the number of discrete service spells, including the one targeted in the study. Finally, the number of subsequent reports investigated during observation refers to the number of any new CPS investigation occurring after the entry in the study and before the end of observation.

### 2.4. Analysis

Bivariate analysis were conducted in two parts. First, placement, kinship care, and stability indicators were compared in the three cohorts. Each cohort is composed of the whole population of children entering CPS during a given time frame. Thus, testing for statistical significance of the differences between the cohorts is not required, since there was no sampling. Second, the median and average cumulative length of stay were computed by type of last permanency attained in each age group in the Post2 cohort. Again, no significance test nor confidence intervals are relevant in this analysis, because all the population under study is included in the cohort.

## 3. Results

The characteristics of the children in the three cohorts and the services they received are given in Table 2 and Table 3. Generally speaking, the children in the Pre cohort can be distinguished from those of the two Post cohorts, whose profiles are fairly similar. So children who entered the CPS system after the reform took effect are younger than those who were taken in before (9.3 years in Pre, as opposed to 8.6 and 8.5 years in Post1 and Post2). Under the new Act, there are proportionally fewer reports classified as neglect, serious behavioral disturbance and abandonment. Physical abuse reports, however, are more frequent. Yet, due to the overhaul of the grounds for protection included in the new provisions of the YPA, it is difficult to compare the relative frequency of the various grounds covered. Four grounds for protection were added under the reform: emotional maltreatment (“psychological ill-treatment”), serious risk of physical abuse, serious risk of sexual abuse and serious risk of neglect. Furthermore, the definition of serious behavioral disturbance eligible for protective measures was formally limited to behavior that repeatedly or seriously undermines the child’s or others’ physical or psychological integrity. Although the safety or development of the vast majority of children in all three cohorts is endangered, since the changes to the Act, there are proportionally fewer children in this situation (Pre: 95%; Post1: 93%; and Post2: 93%). We should recall here that the criteria to be admitted in the study is to be the subject of a CPS investigation during a given timeframe and to receive protective measures during or after this investigation. Thus, the nature of the population under study explain the high percentage of children considered as endangered. On the other hand, a growing minority of children investigated, yet receiving protective measures, had their case closed after investigation because their safety or development was not found to be endangered. Last, the length of services decreased very slightly from one cohort to another, but the number of CPS episodes and the number of subsequent CPS investigations remained quite the same over time (Table 3).

Table 2 also shows that the use of placement has declined slightly in all cohorts. In the Pre cohort, 66% of children had at least one placement in the four years of observation, whereas in Post1, this proportion decreased to 63%, and then 60% in Post2. Among the children in care, a small number in each cohort (*n* = 91 in Pre, 82 in Post1 and 51 in Post2) were only placed intermittently, never continuously. Intermittent placements are very brief placements repeated within a certain period. An example would be children placed with respite foster families every weekend for six months and returning to their own families during the week. Those children are excluded from the following tables.

The use of kinship care increased immediately after the reform and since then has remained stable (Table 4). More precisely, 10% of children in care in the Pre cohort were solely in this type of setting, while that was the case for 17% of children in both Post cohorts. Some children experience a combination of the two types of placement, kinship and non-kinship, and these types of cases increased very slightly from one cohort to another (17%, 18% and 19% in the three cohorts). On the other hand, the proportion of children placed solely in non-kinship care dropped from 73% in Pre to 64% in Post 2.

Table 5 shows the breakdown of the two stability indicators developed for the study. The first notable aspect is that the number of different substitute homes the children stayed in during the four years following their intake into the CPS system declined slightly immediately after the new YPA came into effect and then remained steady. In both Post cohorts, we found proportionally more children who had only one substitute home, compared with the Pre cohort (40% and 41% in Post, as opposed to 36% in Pre). In parallel, there are fewer children with stays in more than three different substitute homes (18% and 17% in Post, against 22% in Pre). The mean number of substitute homes is 2.3 in Post, but 2.5 in Pre. The median number of different substitute homes visited is two in the three cohorts. Looking at moves, Table 5 also shows that the children in the two Post cohorts were moved around slightly less than those in the Pre cohort and the decline was continuous (although minimal) across the three. Thus, in the Pre cohort, 47% of children in care were not moved during the observation period, while the proportion rose to 50% in Post. Similarly, there are slightly fewer children who were moved more than twice in each subsequent cohort (17%, 15% and 13% in Pre, Post1 and Post2, respectively). In the four years following their intake, the children in the Pre cohort were moved 1.3 times on average, while those in the Post1 cohort were moved 1.2 times and those in the Post2 cohort 1.1 times. The median number of moves in cohort Pre and Post1 is one and the median number of moves in cohort Post2 is zero.

Table 6 and Table 7 deal with the latest type of permanency attained, the cumulative length of stay before attainment, and lack of sustainability among children from Post2 cohort whose case went before the courts and who were placed with non-kin during the observation. First, Table 6 shows cumulative length of stay for the various subsets of the cohort. The median cumulative length of stay for the entire cohort is 515 days. Longer stays in care are seen among children aged 6 to 11 at the time of their first placement, while the shortest are observed for children aged age 12 and more.

The cumulative lengths of stay for each type of permanency are reported by age at time of first placement (Table 7). Among children under 2, the type of permanency most often attained by the end of observation was adoption, followed by placement until the age of 18 and then reunification. More precisely, among the youngest children, those whose latest permanency attained was adoption had a median stay of 699 days in substitute care before being adopted. Half of those placed until 18 accumulated 548 days in care before a court order was issued. The proportion of children in care until 18 years old who were moved after a court order was 7%. Children reunited with their families spent 318 days in substitute care and 17% were placed again after reunification. Among 2- to 5-year-olds, the type of permanency most often attained was reunification, followed by placement until the age of 18. Half of these children spent 310 days in substitute care before being reunited with their families and 16% were placed again after reunification. Those placed in care until 18 spent 647 days in substitute care before the court order and none was moved after the order. Children whose latest type of permanency attained was adoption spent a mean total of 874 days in substitute care before being adopted. Among 6- to 11-year-olds, the type of permanency most often attained was reunification, followed by placement until the age of 18. Those who were reunited with their families spent 385 days in substitute care and 24% were placed again after reunification. Children placed until 18 spent 738 days in care before the court order and 14% were moved after the order. Among 12- to 17-year-olds, the type of permanency most often attained was reunification, followed by placement until the age of 18. Those who were reunited with their families spent 377 days in substitute care and 19% were placed again after reunification. Children placed until 18 spent 646 days in care before the court order and 38% were moved afterwards.

Four observations may be made with respect to these findings on length of stay, permanency, and sustainability. First, reunification is the type of permanency with the shortest cumulative length of stay (310 to 385 days, depending on the age group). However, a considerable proportion of children reunited with their families were placed in care again within the observation period (between 17% and 24%, depending on the age group). Second, a significant proportion of children do not seem to have exited to permanency within the study period. This is especially true of children aged 2 to 5 (29%) and 6 to 11 (25%). Furthermore, these children spent a long time in out-of-home care during the observation period: a median of 713 days and 767 days in these two age groups. Third, the length of stay is underestimated because kinship care is not counted, although some of the children did experience this kind of placement. The percentage of children placed with kin at least once in the observation period varies between 15% and 40%. Fourth, the permanency attained that was used to calculate cumulative length of stay in care was not, for some children, the only attempt for permanency. Unsurprisingly, the number of exits to permanency during the observation period was higher for adolescents (mean of 1.6 to 2.3, depending on the latest type of permanency attained), while for the youngest, the mean oscillated between 1.1 and 1.5.

## 4. Discussion

Based on three populations of children entering CPS across the province of Quebec, this study describes a number of aspects of the placement trajectory of children receiving child protective services. It enabled us to reassess changes in the use of out-of-home care, the use of kinship care, and stability since the reform, this time examining a longer observation period and a more recent cohort. In addition, we established a preliminary profile of stays in foster care by age and type of exit to permanency. The findings demonstrate encouraging but marginal trends with regard to the use of substitute care and stability, as well as the fact that the reform has not had any unintended harmful consequences. They also raise some important questions about the degree to which some life plans do not result in permanency and highlight some situations of concern that require monitoring.

### 4.1. Placement, Kinship Care and Stability

The frequency of placement and the type of substitute care prioritized by CPS have changed very little since the overhaul of the Act. The use of out-of-home care declined slightly immediately after the new provisions came into effect and continued in the following years. In Quebec, when children must be removed from their homes, CPS now opt for kinship care more often. These changes in placements and in the choice of living environment are in line with the principles set out in the legislative provisions that came into effect in 2007. The lower frequency of placement of children in substitute care (along with the changes in other aspects of the placement trajectory) is probably partly attributable to the change in the profile of children reported to CPS. Since the overhaul of the Act, there have been proportionally fewer children between the ages of 12 and 17 under CPS supervision, and fewer children being supervised because of serious behavioral disturbances. After abandonment, serious behavioral disturbances are the most common ground for placing children in substitute care in Quebec [5]. In addition, this decline in the proportion of teens under CPS supervision and the proportion being followed due to behavioral problems might also explain, at least partly, the greater proportion placed in kinship care. That is because teens with behavioral problems are rarely placed with kin [5].

Quebec children in care typically experience one move and two different substitute homes over a period of three to four years after intake. The extent of this instability is consistent with that reported in a study done in England with a 3.5-year observation period [34]. Changes in stability took place immediately after the reform and the situation has remained the same since then. Each of the two instability indicators considered here declined, although the magnitude of the decline was small. These findings concerning instability are about the same as those obtained in the first assessment of the YPA, despite the fact that the observation period of the first assessment was half as long, which suggests that instability is more likely within the first two years that children are under CPS supervision. Stability would need to be measured over a very long period of time to confirm this hypothesis. Further examination of the different patterns of placement should be conducted. In this regard, one study propose patterns of placement which take into account the timing of placement moves as well as the movement along degree of restrictiveness of the type of placement setting [35].

### 4.2. Cumulative Length of Stay, Type of Permanency, and Sustainability

Our study paints a preliminary picture of cumulative length of stay in substitute care by children who exited to permanency, in the form of family reunification, placement until 18, and adoption. Reunification and placement until 18 were the most common options for most age groups. Adoption was elected exclusively for children who are younger at the time of placement. In this study, only 7% of children of all ages were adopted during the three or four years of observation. In comparison, in other jurisdictions, the adoption rate varied between 9% and 25% in observation periods of 19 to 48 months [16,43], and reached 34% over 6 years [51]. It is possible that Quebec caseworkers and courts are reluctant to favor adoption as a permanent plan for school-aged children and teens who have already formed attachment bonds with their birth parents. Placement until the age of 18 could then be the alternative chosen for these older children who cannot go back to their families. Also, unlike the American Adoption and Safe Families Act, Quebec’s Youth Protection Act does not provide for termination of parental rights after children have spent a certain amount of time in substitute care, so children placed in care until they turn 18 can maintain a connection to their biological parents. This may as well be the case for youth who end up leaving care for independent living.

The frequency of family reunification and cumulative length of stay in care before reunification is attained are within the range of measurements reported in earlier studies with similar observation periods. Thus, in 53% of cases, reunification was preferred and typically took place 9 to 12 months after placement in care, which is consistent with the findings of several studies [43,52,53], and with more recent Quebec findings [37]. Reunification was the option tried soonest. This is in line with one of the principles of Quebec’s Youth Protection Act, which is to keep children in the family environment unless it is not in their best interests. Yet a far from negligible proportion of children returned to their homes were taken into care again within three or four years of entering the CPS system (between 16% and 24%, depending on their age). Reentry rates reported here are consistent with those in other studies with similar observation periods. For example, one study found a reentry rate of 22% within the six years following initial placement [54]. Overall, these findings provide evidence of the challenges associated with returning children to their families after out-of-home care and the importance of supporting parents and children at reunification. Moreover, according to earlier studies, children’s personal problems (substance abuse, behavioral disturbances, learning difficulties), parents’ problems (criminality, substance abuse), as well as living conditions at the time of reunification (poverty, overcrowding, homelessness) are associated with an increased likelihood of reunification breakdown [47,54,55,56,57]. Yet these studies say little about the kinds of clinical activities carried out in the weeks surrounding reunification that might increase the odds of success. Greater familiarity with such clinical activities could help determine which services are more effective at keeping families reunified. Reunification breakdown may also be due to the difficulty that front-line services have in picking up where CPS casework leaves off. For this reason, formal collaboration protocols between child protective services and front-line social services should be developed.

We found that over a third of adolescents placed in care until the age of 18 were moved from one substitute home to another. In such a situation, the preferred permanency option does not achieve the stated goal of the Act: to provide “continuity of care, stable relationships and stable living conditions”. Adolescence may present special challenges to teens not living with their families. So it is essential, first, to provide better assistance to teens in care and to better equip their foster homes during this period, and second, to better document the problems so as to be able to implement measures that will help ensure greater stability for these youth.

It’s interesting to note that for some children, cumulative time in care is far above the age-specific maximum durations of stay that is prescribed by the new dispositions of the YPA. This is especially the case for children aged less than 2 years-old and, to a lesser extent, for children aged 2 to 11. However, the length of stay calculated here includes placements in service spells prior to the one targeted by the study. But, under the new provisions of the YPA, the courts are not required to count placements during earlier service spells to determine the permanency plan; that is left to the judge’s discretion. Prior analysis with the current cohorts indicate that when placements occurring in a previous service spell are excluded from the calculation of the length of stay in care, the duration of stay is still overridden for adoption and placement until majority for children aged less than 12 [58]. This observation raises the question of operationalization of a cumulative length of stay that truly reflects children’s out-of-home care experience. The challenges are both clinical and methodological: Why would placements occurring during prior service spells not be considered to guide the clinical decisions about the best permanency plan for a child?

During the observation period, there was a significant percentage of children in care who have not exited care within the three or four years of their intake into the CPS system. This is particularly true of children aged 2 to 11 at the time of placement covered in the study. An annotated review of Quebec case law found that in some situations, although a placement is not planned to last until the child turns 18, the court has actually ordered it with a view to permanency [59]. More precisely, according to Charrette, “Over the years, the courts have affirmed in some rulings that a permanent order did not necessarily mean until the age of majority, although conversely, a few decisions concluded that only such an order could be considered permanent” (p. 38). In light of this analysis, it is possible that in the current study, some “permanent” plans were not actually coded as permanent. It is necessary to better document the situation of these children in order to understand what it is that characterizes the situations of these children and identify the reasons why they have—at least apparently—not attained permanency.

### 4.3. Strengths and Limitations

The lengths of stay calculated in this study are conservative estimates of the actual time children have spent in substitute care in their lifetimes. At the time the data were extracted, placements in kinship care were not documented as accurately as placements with formal CPS resources, so we could not include days spent with kin in the calculation of length of stay in care. Yet between 15% and 40% of children, depending on their age, spend time in kinship care.

The current evaluative study cannot attribute the differences observed between the cohorts to the new provisions of the YPA with certainty. To do so, a number of aspects associated with organizational settings or regional services for youth in difficulty, among other things, would need to be systematically controlled for. Furthermore, the observed differences in the composition of the cohorts might partly explain the changes in the aspects being studied. Multivariate techniques, such as Cox regression models, may be undertaken to identify determinants of placement trajectory indicators, while controlling for sociodemographic and case characteristics as well as unequal duration of follow-up between children. Work is currently underway in this regard.

Last, this study is based solely on clinical and administrative data from the 16 Quebec CPS agencies. This data source is both a strength and a limitation. On the one hand, the use of clinical and administrative data allows the entire population concerned to be covered without the need for sampling procedures, thus maximizing the reliability and representativeness of the results. In addition, using clinical and administrative data means that the dimensions being studied can be measured repeatedly without having to gather data from clients or caseworkers a second time, which certainly makes it easier to do later studies. On the other hand, the validity of the study results depends on the quality of the data entered in the client information system. Great efforts have been made to standardize both processes and content of this system throughout Quebec, and accordingly the vast majority of data contained by the system is considered to be of very good quality. However, some data have limited potential for analysis because there is no systematic continuous input. These special aspects had to be taken into account in the analyses, for one thing, by excluding kinship care from the stability indicators, length of stay, and type of permanency. As a result, the indicators created are conservative estimates of instability and cumulative length of stay in care. Also, as seen in administrative data from other parts of the world, there is a great deal of some clinical information, but it cannot be used on a large scale because of how it was entered. That is the case for children’s individual problems and case characteristics, psychosocial characteristics of parents and substitute homes, quality of relationships between parents and out-of-home care providers, and clinical activities carried out to protect the child. These dimensions are documented in various clinical reports which are entered in the system, but as the reports are full text, analysis demands a qualitative approach that is very time-consuming and not always feasible. This wealth of clinical data lying dormant in the information systems would nevertheless be well worth exploring. Taking into account such clinical dimensions in future studies would make it possible to better qualify children’s experiences of substitute care and provide guidance for programs and policies aiming to give them stability. In this sense, the third YPA assessment cycle that is getting under way now [60] will combine the extraction of administrative data with a reading of clinical files for a sample of children reunited with their families, in order to document the factors associated with making reunification successful.

## 5. Conclusions

This second study on the overhaul of the Quebec Youth Protection Act reveals that in the medium term, changing patterns in out-of-home placement, kinship care, and stability are aligned with the goals of the YPA, although the changes are quite small in magnitude. Future assessment cycles will enable an evaluation of changes in the types of permanency and cumulative length of stay in substitute care; in this study, however, the figures were available for only one cohort. This study has also identified subsets of children whose situation is of greater concern in terms of stability and living conditions. Work on the third YPA study has already begun. This time, the focus will be on family reunification and the factors associated with its breakdown.

## Figures and Tables

**Table 1:** Eligibility period and observation period for each cohort.

Cohort	Eligibility Period	Observation Period
Pre	1 July 2003–30 June 2004	1 July 2003–30 June 2007
Post1	1 September 2007–31 August 2008	1 September 2007–31 August 2011
Post2	1 January 2009–31 December 2009	1 January 2009–31 December 2012

**Table 2:** Children and case characteristics—Categorical variables.

Children and Case Characteristics-Categorical Variables		Pre	Post1	Post2
		*n* = 9620	*n* = 8676	*n* = 8425
Placement		6387, 66.4%	5449, 62.8%	5019, 59.6%
Age group at entry in cohort	<2 years	1088, 11.3%	1412, 16.3%	1452, 17.2%
	2–5 years	1525, 15.9%	1460, 16.8%	1448, 17.2%
	6–11 years	2837, 29.5%	2403, 27.7%	2258, 26.8%
	12–17 years	4169, 43.3%	3399, 39.2%	3261, 38.7%
Gender	Boys	5013, 52.1%	4553, 52.5%	4435, 52.6%
	Girls	4606, 47.9%	4122, 47.5%	3990, 47.4%
Indigenous		533, 5.5%	578, 6.7%	485, 5.8%
Grounds for intervention	Neglect	7699, 80.0%	4981, 57.4%	5164, 61.3%
	Serious risk of neglect *	380, 4.0%	4400, 50.7%	4179, 49.6%
	Physical abuse	2072, 21.5%	2615, 30.1%	2670, 31.7%
	Serious risk of physical abuse *	40, 0.4%	1361, 15.7%	1430, 17.0%
	Sexual abuse	1400, 14.6%	1165, 13.4%	1028, 12.2%
	Serious risk of sexual abuse *	18, 0.2%	873, 10.1%	764, 9.1%
	Serious behavioral disturbance	4595, 47.8%	3194, 36.8%	3102, 36.8%
	Abandonment	798, 8.3%	302, 3.5%	250, 3.0%
	Emotional maltreatment *	218, 2.3%	4136, 47.7%	4167, 49.5%
Conclusion of investigation	Founded allegations, safety or development endangered	9145, 95.1%	8067, 93.1%	7854, 93.4%
	Founded allegations, safety or development not endangered	307, 3.2%	430, 5.0%	399, 4.7%
	Unfounded allegations	153, 1.6%	149, 1.7%	146, 1.7%
	Other conclusion	11, 0.1%	19, 0.3%	14, 0.1%
Court use		6691, 69.6%	6130, 70.7%	5904, 70.1%

* Ground for intervention added in the new Act.

**Table 3:** Children and case characteristics—Continuous variables.

Children and Case Characteristics-Continuous Variables	Pre	Post1	Post2
Age at entry in cohort (years)	9.3 (0–17)	8.6 (0–17)	8.5 (0–17)
Length of service (months)	25.7 (0–48)	25.3 (0–48)	25.0 (0–48)
Number of subsequent reports investigated during observation	0.7 (0–9)	0.6 (0–8)	0.6 (0–8)
Number of CPS episodes during observation	1.1 (1–3)	1.1 (1–4)	1.1 (1–3)

**Table 4:** Type of placement settings among children placed continuously.

Type of Placement Settings	Pre	Post1	Post2
	*n* = 6296 (%)	*n* = 5367 (%)	*n* = 4968 (%)
Non-kinship only	4568, 72.6%	3480, 64.8%	3187, 64.2%
Kinship and non-kinship	1078, 17.1%	971, 18.1%	944, 19.0%
Kinship only	650, 10.3%	916, 17.1%	837, 16.8%

**Table 5:** Stability of children placed continuously, with at least one placement in non-kinship care.

Stability of Children Placed in Care		Pre	Post1	Post2
		*n* = 5646 (%)	*n* = 4451 (%)	*n* = 4131 (%)
Number of different substitute homes	One	2043, 36.2%	1799, 40.4%	1697, 41.1%
	Two	1500, 26.6%	1182, 26.6%	1139, 27.6%
	Three	880, 15.6%	653, 14.7%	614, 14.9%
	More than three	1223, 21.7%	817, 18.4%	681, 16.5%
	Mean (S.D.)	2.5 (1.7)	2.3 (1.6)	2.3 (1.6)
	Median	2	2	2
	Min–Max	1–13	1–13	1–13
Number of moves	None	2634, 46.7%	2203, 49.5%	2074, 50.2%
	One	1381, 24.5%	1075, 24.2%	1056, 25.6%
	Two	692, 12.3%	501, 11.3%	456, 11%
	More than two	939, 16.6%	672, 15.1%	545, 13.2%
	Mean (S.D.) ^(1)^	1.3 (1.9)	1.2 (2)	1.1 (1.8)
	Median	1	1	0
	Min–Max ^(1)^	0–19	0–19	0–18

^(1)^ 17 children have 20 moves or more and are excluded (Pre: 4, Post1: 6, Post2: 7).

**Table 6:** Cumulative length of stay among children from cohort Post2 placed at least once in non-kinship care and whose case have been brought to court (*n* = 2106).

Children Characteristics		Cumulative Length of Stay (Days)		
		Mean	S.D.	Median
Age group at first placement	<2	620.7	307.1	583.5
	2–5	584.9	331.5	563.0
	6–11	627.6	380.2	611.0
	12 and more	556.1	423.3	449.5
Gender	Boys	586.2	391.4	509.0
	Girls	578.1	387.5	518.0
Total		582.61	389.63	514.5

**Table 7:** Type of permanency and cumulative time in care according to the age of the child at first placement, among children from cohort Post2 who are placed at least once in non-kinship and whose case have been brought to court (*n* = 2106).

Age at First Placement	Last Type of Permanency Attained			Cumulative Length of Stay in Care (Days)		Kinship Care	Lack of Sustainability	Number of Exits to Permanency
		*n*	%	Mean (S.D.)	Median	%	%	Mean
<2	Reunification	84	24.2	389.9 (238.2)	318.0	27.4	17.3	1.1
	Adoption	127	36.6	755.9 (264.8)	699.0	18.1		1.4
	Until majority	97	28.0	588.8 (245.3)	548.0	17.5	7.1	1.3
	None	39	11.2	777.1 (380.1)	844.0	17.9		
2–5	Reunification	90	38.8	374.1 (217.7)	310.0	40.0	15.9	1.1
	Adoption	13	5.6	894.5 (270.2)	874.0	15.4		1.5
	Until majority	62	26.7	677.6 (255.3)	646.5	29.0	0.0	1.3
	None	67	28.9	731.8 (378.5)	713.0	32.8		
6–11	Reunification	165	46.3	453.0 (307.4)	385.0	26.7	23.8	1.2
	Adoption	1	--	--	--	--		--
	Until majority	102	28.7	790.9 (313.7)	737.5	20.6	13.5	1.2
	None	88	24.7	752.1 (442.0)	767.0	25.0		
12–17	Reunification	750	66.2	476.0 (377.3)	377.0	15.3	19.4	1.6
	Majority	205	18.1	721.6 (481.0)	646.0	20.5	37.8	1.7
	None: Independent living	81	7.1	690.6 (332.5)	676.0	17.3	15.9	2.3
	None: other	97	8.6	705.2 (535.0)	589.0	20.6		
Total	Reunification	1089	52.7	457.4 (348.7)	366.0	20.0	19.6	1.5
	Adoption	141	6.8	767.6 (266.8)	715.0	18.4		1.4
	Until majority	466	22.5	703.3 (385.3)	647.0	21.0	21.6	1.4
	None: Independent living	81	3.9	690.6 (332.5)	676.0	17.3	15.9	2.3
	None: other	291	14.1	735.2 (453.3)	688.0	24.4		
